# Beyond the surface: Advancing neurorehabilitation with transcranial temporal interference stimulation — clinical applications and future prospects

**DOI:** 10.4103/NRR.NRR-D-24-01573

**Published:** 2025-03-25

**Authors:** Camille E. Proulx, Friedhelm C. Hummel

**Affiliations:** Defitech Chair of Clinical Neuroengineering, Neuro-X Institute (INX), Swiss Federal Institute of Technology (EPFL), Geneva, Switzerland; Defitech Chair of Clinical Neuroengineering, INX, EPFL Valais, Clinique Romande de Réadaptation (CRR), Sion, Switzerland; Clinical Neuroscience, University of Geneva Medical School, Geneva, Switzerland

**Figure 1:**
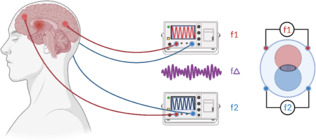
tTIS human experimental montage and the underlying concept. The concept of tTIS for brain stimulation was first suggested through computational modeling and preclinical experiments in mice (Grossman et al., 2017). This technique involves the use of two pairs of electrodes positioned on the surface of the scalp to deliver two high-frequency (e.g., > 1 kHz) transcranial electrical carrier currents that are slightly offset in frequency (f), resulting in the generation of a low-frequency envelope, an amplitude modulated envelope. This envelope selectively interacts with the neural membranes of brain tissue sensitive to low-frequency electrical signals, while high-frequency currents remain physiologically inactive. By modifying electrode configuration or adjusting current parameters, the location of the peak envelope amplitude can be precisely steered to target specific deep brain structures, such as the basal ganglia and the hippocampus (Wessel et al., 2023; Beanato et al., 2024; Vassiliadis et al., 2024). Created with BioRender.com. tTIS: Transcranial temporal interference stimulation.

Brain lesions, such as those caused by stroke or traumatic brain injury (TBI), frequently result in persistent motor and cognitive impairments that significantly affect the individual patient’s quality of life. Despite differences in the mechanisms of injury, both conditions share a high prevalence of motor and cognitive impairments. These deficits show only limited natural recovery. Therefore, the impairments following brain injury mandate better treatment and represent critically important targets for novel interventional strategies in neurorehabilitation. By focusing on improving recovery and promoting functional independence, neurorehabilitation plays a crucial role in helping individuals regain the ability to perform daily life activities, addressing the growing global burden of neurological disorders. Non-invasive brain stimulation has emerged as a promising approach to neurorehabilitation, with its capacity to modulate activity in targeted brain regions. Techniques such as transcranial direct current stimulation and transcranial magnetic stimulation (TMS) are among the most extensively studied, recognized for their potential to influence cortical activity and induce changes across motor, sensory, and cognitive networks. While these methods are considered safe compared to invasive alternatives, they have the limitation of only effectively targeting cortical brain regions. Considering the critical role, supported by imaging studies and animal models, of subcortical brain areas, such as the striatum, thalamus, or hippocampus for motor and cognitive processes, there is a growing need for non-invasive approaches capable of reaching deeper brain structures (Miyachi et al., 1997). Currently, a novel, promising, innovative, and safe neuromodulation technique called transcranial temporal interference stimulation (tTIS) has been introduced (**Figure 1**), with the potential to address these challenges by selectively targeting deeper brain regions without impacting overlying structures (Wessel et al., 2023; Beanato et al., 2024; Vassiliadis et al., 2024). As a novel approach, it is not surprising that research on its application in human neurorehabilitation remains limited. This manuscript seeks to provide an overview of tTIS in the context towards application in neurorehabilitation, particularly for stroke and TBI, to address current challenges, and, most importantly, to provide a forward-looking perspective on its future clinical applications and transformative impact on the field. Additionally, it highlights emerging areas of research and upcoming studies that promise to advance our understanding and implementation of this innovative technology.

**Exploring the clinical potential of transcranial temporal interference stimulation of subcortical regions for neurorehabilitation:** The basal ganglia, in particular the striatum, are strongly involved in motor control, motor learning, and acquisition of new skills. Miyachi et al. (1997) investigated the role of specific striatal regions by inactivating them in non-human primates and assessing the impact on the acquisition of new sequences and the execution of well-learned ones. Their study highlighted the dorsal striatum’s critical involvement in both sequence learning (motor memory) and performance, making it a prime target for neuromodulation, which ultimately led to the exploration of tTIS with a focus on targeting the striatum to enhance motor performance and learning for the first time in humans (Wessel et al., 2023). When applied to the striatum of healthy humans, tTIS significantly enhanced motor task performance, accompanied by modulation of striatal activity in the putamen, as confirmed by blood oxygen level–dependent activity changes. This effect was observed when compared to a high-frequency control stimulation condition without a frequency difference, demonstrating the potential of tTIS to specifically engage and modulate striatal regions involved in motor control. These findings are particularly relevant given the well-documented challenges in motor sequence learning caused by subcortical brain lesions affecting the basal ganglia in stroke patients. Such lesions result in significantly reduced performance improvements in motor sequence-specific learning tasks compared to healthy controls, emphasizing the critical role of the basal ganglia in this population, and their potential as a target for tTIS.

Another key subcortical region involved in the performance of goal-directed tasks, and sharing functional connectivity with the striatum, but mostly in support of cognitive functions, is the hippocampus. This region plays a crucial role in spatial navigation and episodic memory and emerges as a promising target for tTIS, particularly given its demonstrated benefits in healthy populations. For instance, tTIS applied in an intermittent theta-burst stimulation pattern (iTBS) — inducing long-term potentiation-like effects within the hippocampal-entorhinal complex has been shown to enhance performance in a virtual spatial navigation task (Beanato et al., 2024). Specifically, improvements included faster object position recall without diminished accuracy and increased hippocampal blood oxygen level–dependent activity. Beyond spatial navigation, Violante et al. (2023) further demonstrated, through fMRI and behavioral experiments, the effect of tTIS in modulating hippocampal activity and enhancing episodic memory in healthy individuals. These studies provide essential proof-of-concept evidence of the effects of tTIS on cognitive functions in healthy populations, highlighting its potential for addressing cognitive impairments commonly observed in stroke and TBI populations. The relevance and promise of hippocampal tTIS in improving these cognitive functions are particularly compelling.

**Current limitations, future directions, and upcoming studies:** As tTIS progresses from animal studies to human applications, several critical gaps must be addressed before this technology can be effectively translated into clinical practice to improve patients’ daily lives. These critical challenges will be presented hereunder and have to be addressed in the upcoming years.

***Clinical application of transcranial temporal interference stimulation protocols:*** Despite no published rehabilitation studies yet, several clinical trials are ongoing to demonstrate the promising potential benefits of striatal and hippocampal tTIS for improving motor and cognitive functions in impaired populations, such as stroke and TBI. For instance, a recently shared preprint is outlining a clinical trial protocol investigating the effects of hippocampal tTIS on cognitive functions in stroke patients (Maimaitiali et al., 2024). Such clinical trials are crucial for advancing tTIS toward clinical application, as they aim to establish its efficacy and optimize therapeutic outcomes in neurorehabilitation. Drawing from established approaches in other patient populations, where non-invasive brain stimulation has already been successfully integrated into clinical practice, could provide valuable insights for designing and implementing innovative and effective tTIS protocols. A notable example is the recent FDA approval of the Stanford Accelerated Intelligent Neuromodulation Therapy protocol for high-dose, accelerated iTBS in individuals with psychiatric disorders. This protocol represents a significant advancement in the field, leveraging a condensed stimulation approach by delivering multiple iTBS sessions within a single day, guided by the principle of “spaced learning/training”—the concept that distributed repetition with intervals is more effective for learning or training than a single session of massed repetition. Specifically, Stanford Accelerated Intelligent Neuromodulation Therapy condenses six weeks of traditional daily rTMS sessions into 10 daily sessions of iTBS spaced 50 minutes apart, administered over 5 consecutive days. For example, this safe and well-tolerated approach has been shown to be associated with significant reductions in depressive symptoms and suicidal ideation in patients with treatment-resistant depression, and higher remission rates compared to standard rTMS protocols. These advancements highlight the potential for developing similarly innovative, accelerated protocols tailored specifically for tTIS, designed to address motor and cognitive impairments in neurological populations such as those with stroke and TBI, and to validate them.

***Individualized approach:*** In addition to optimizing the experimental design for clinical protocols, there is also a need for the optimization of stimulation parameters. As it is now widely recognized, there is no one-size-fits-all approach in neurorehabilitation, as various factors critically influence outcomes. An individual’s unique anatomy, both structural and functional connectomics, along with lesion-induced network changes, each play a distinct role in shaping the brain’s responsiveness to clinical neuromodulation protocols. In that sense, efforts are being made to further individualize protocols through biomarker (e.g., clinical, behavioral, TMS, MRI) stratification, which aims to tailor stimulation parameters to specific neurophysiological profiles. In the context of tTIS, computational modeling allows for the optimization of electrode configurations tailored to these unique biomarkers. For instance, to achieve greater individualized stimulation focality, a genetic algorithm can be applied to optimize electrode placement and current intensity by maximizing the volume-weighted electric field ratio between the region of interest and surrounding non-region of interest gray matter areas (Stoupis and Samaras, 2022). Furthermore, based on brain activity patterns, it enables the refinement of stimulation parameters such as phase, amplitude, and frequency intensities, which holds the transformative potential to maximize its effects. However, despite its utility, this computational modeling approach remains limited in its ability to deliver truly personalized interventions. Indeed, the field is hindered by the absence of robust and powerful predictive models capable of reliably guiding these individualized interventions (Wessel et al., 2021). A promising solution to this challenge lies in the adoption of online optimization methods. For instance, a Gaussian-process-based Bayesian optimization algorithm has been shown to successfully optimize, in-real time, the efficacy of neurostimulation in animal models (Choinière et al., 2024). That said, a critical limitation from this method currently lies in the absence of a “proxy” to directly measure the real-time effects of tTIS and provide online feedback on its efficacy. For instance, EEG could hold promise as a potential proxy for tTIS due to its ability to monitor real time neural activity. In return, EEG can only record cortical activity thus only indirect factors that are driven by deep structures such as the striatum, hippocampus, or thalamus. Also, no published study yet has concurrently employed both technologies to explore this possibility. Encouragingly, preliminary efforts are underway, with research teams actively investigating this approach. Addressing this gap is essential to fully unlock the potential of tTIS and other neurostimulation methods in personalized neurorehabilitation, where adaptive and precise interventions could significantly improve therapeutic outcomes.

***Transcranial temporal interference stimulation to enhance ecological functions:*** Currently, the effects of tTIS are primarily tested in controlled laboratory settings using standardized and relatively simple tasks, such as gripping or finger-tapping tasks. While these provide valuable insights, they fail to capture the complexity and multifaceted nature of real-world activities. The dynamic interaction of motor, cognitive, and contextual factors that characterise everyday life activities is not adequately represented in these simplified experimental tasks. Given that tTIS has been shown to effectively neuromodulate brain activity, solely when stimulation is applied during task-evoked activity (Wessel et al., 2023), current tTIS equipment hinders its broader application in daily life. Specifically, tTIS requires the use of two quite bulky and heavy stimulators necessitating their placement on a stable surface near the subject to ensure proper functionality. This requirement restricts the subject’s movements, limiting the types of tasks they can perform during stimulation. Addressing this limitation on a hardware side will be crucial for bridging the gap between laboratory findings and practical, clinically relevant applications of tTIS. Improving the portability of the equipment is crucial, as it would allow for use in more natural, everyday environments, such as at home. In the meantime, an alternative approach to enhance ecological validity involves integrating virtual reality elements into task design. For example, neuromodulation of the hippocampal-entorhinal complex during a virtual reality-based spatial navigation task serves as a promising example of how more realistic conditions for assessment can be created to determine the effects of non-invasive deep brain stimulation (Beanato et al., 2024). This direction offers a promising avenue for future research, particularly as it helps overcome current hardware limitations and paves the way for more ecological, non-laboratory-based applications of tTIS.

**Figure 2:**
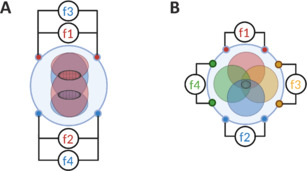
Multipoint tTIS and multipolar tTIS underlying concept. (A) The multipoint technique involves two pairs of electrodes delivering four electrically isolated currents, each at a distinct frequency. These currents interfere in pairs at specific target regions, producing two distinct AM envelopes. (B) The multipolar technique involves the use of multiple pairs of electrodes delivering multiple currents at distinct frequencies. These currents interfere in pairs, generating multiple AM envelopes that further interact to form a single focal envelope at a specific location. Created with BioRender.com. AM: Amplitude modulated; tTIS: transcranial temporal interference stimulation.

***Innovative technical approach in transcranial temporal interference stimulation:*** At last, there are also technical innovative approaches that merit exploration in the coming years. These techniques include multipoint tTIS (Zhu et al., 2019) and multipolar tTIS (Acerbo et al., 2024), which enables two distinct goals — respectively, the stimulation of multiple sites simultaneously with only two pairs of electrodes or the enhancement of stimulation’s focality at one specific location with multiple pairs of electrodes (**Figure 2**). Building on these foundations, this article proposes clinical examples of how these techniques could advance the field of neurorehabilitation. Given, the well-documented interconnected roles of the striatum and hippocampus in motor learning and memory, exploring the concomitant stimulation of these two regions through multipoint tTIS presents an exciting avenue for research. This approach could unlock new possibilities in targeting the integrated neural circuits underlying these complex cognitive and motor processes. On the counterpart, the inherent complexity of regions such as the striatum and hippocampus — where different subregions are specialized for distinct functions (e.g., the role of the dorsal striatum in motor processes and the involvement of the ventral striatum in motivation) — underscores the potential of multipolar tTIS. By improving the precision of stimulation, multipolar tTIS allows for the selective targeting of specific subregions, potentially enhancing the efficacy and therapeutic outcomes of neuromodulation interventions in clinical populations.

**Conclusion:** This article underscores the promising, transformative potential of non-invasive deep brain stimulation, particularly tTIS, for the field of neurorehabilitation. Recent advances highlight its ability to modulate striatal and hippocampal activity, demonstrating improvements in motor and cognitive functions in healthy populations. These findings lay a strong foundation for the translation of tTIS into initial clinical applications for populations with stroke and TBI, as reflected by the flow of studies currently underway. Moreover, the coming years, through collaborative efforts within the scientific community, will bring exciting opportunities to further optimize the efficacy and feasibility of tTIS in clinical settings. Such advancements have the potential to make significant strides toward the ultimate goal of improving the quality of life of individuals with brain injuries.

*We acknowledge BioRender for providing the platform used to create the figures included in this manuscript - Figure 1. https://BioRender.com/c90v791; Figure 2. https://BioRender.com/v29q402.*

*This work was supported by the Defitech Foundation (Morges, CH) to FCH; the Bertarelli Foundation -Catalyst program (Gstaad, CH) to FCH; the Wyss Center for Bio and Neuroengineering the Lighthouse Partnership for AI-guided Neuromodulation to FCH; the Fonds de recherche du Québec – Santé (FRQS#342969) to CEP and the Neuro X Postdoctoral Fellowship Program to CEP.*
